# 1149. Application of a Multiplex Polymerase Chain Reaction Test for Diagnosing Bacterial Enteritis in Children in a Real-Life Clinical Setting

**DOI:** 10.1093/ofid/ofab466.1342

**Published:** 2021-12-04

**Authors:** Hyun Woo Lee, Seung Beom Han, Jung-Woo Rhim

**Affiliations:** 1 Daejeon St. Mary’s Hospital, Daejeon, Taejon-jikhalsi, Republic of Korea; 2 College of Medicine, The Catholic University of Korea, Daejon, Taejon-jikhalsi, Republic of Korea

## Abstract

**Background:**

Although a bacterial multiplex polymerase chain reaction (mPCR) test should be performed selectively in patients with gastrointestinal symptoms consistent with bacterial enteritis, its usefulness has been evaluated upon stool samples as requested by clinicians, without considering the patients’ gastrointestinal symptoms or clinical diagnoses. This study aimed to determine the subjects to bacterial mPCR testing and to interpret the mPCR test results with considering patients’ clinical symptoms and diagnoses.

**Methods:**

Medical records of 710 pediatric patients for whom a bacterial mPCR test was performed were retrospectively reviewed. Clinical characteristics and mPCR test results were compared between patients with positive mPCR test results (*n* = 199) and those with negative mPCR test results (*n* = 511) and between patients in whom inflammatory pathogens (*Campylobacter* spp. and *Salmonella* spp.) were identified (*n* = 95) and those in whom toxigenic pathogens (*Clostridium* spp.) were identified (*n* = 70).

**Results:**

A positive mPCR test result was significantly associated with an older age (*p* < 0.001), diagnosis of acute gastroenteritis (*p* = 0.021), presence of hematochezia (*p* < 0.001), and absence of cough (*p* = 0.004). The diagnosis of acute gastroenteritis (*p* = 0.003), presence of fever (*p* = 0.027) and diarrhea (*p* = 0.043), and a higher C-reactive protein level (*p* = 0.025) were significantly associated with the identification of inflammatory pathogens rather than toxigenic pathogens in patients with positive mPCR test results.

**Conclusion:**

Bacterial mPCR testing should be performed selectively based on patients’ clinical symptoms and diagnoses, and its results should be interpreted with considering identified pathogens.

**Disclosures:**

**All Authors**: No reported disclosures

